# Research on Xiaoyao Powder in the treatment of depression based on epigenetics and quality markers

**DOI:** 10.3389/fnins.2023.1223451

**Published:** 2023-08-24

**Authors:** Jiayun Wang, Qiaolan Wu, Chunxue Ou, Guangying Lu, Huayun Yu

**Affiliations:** Shandong University of Traditional Chinese Medicine, Jinan, Shandong, China

**Keywords:** Xiaoyao Powder, depression, clinical application, pharmacological mechanisms, epigenetics, quality markers

## Abstract

Depression has become one of the most common public health issues around the world, and the incidence has been increasing in recent years. A large amount of clinical investigations have proven that the treatment of depression is difficult. The prognosis is poor, and the fatality rate is high. At present, western medicine is the preferred treatment for depression, but it often causes adverse clinical reactions such as dry mouth, blurred vision, and memory loss, etc. The herbal compound Xiaoyao Powder is a traditional medicine for soothing the liver and relieving depression, strengthening the spleen, and nourishing the blood. It can reduce adverse reactions. It is effective in treating depression. In this study, we elucidate the function of Xiaoyao Powder in anti-depression from the perspective of clinical application and pharmacological mechanisms such as regulating epigenetic and chemical quality markers to provide empirical and experimental theoretical results that contribute to developing future depression therapy with Xiaoyao Powder.

## Introduction

Depression is a type of emotional mental disorder that harms human physical and mental health. Studies have shown that morbidity in men is lower than in women (Ivanets et al., 2021). With the increasing pressures of modern society, emotional disorders and cognitive impairments are on the rise in the global burden of disease, and have received widespread attention (Huang et al., 2019; Perini et al., 2019). In clinical medication, 5-hydroxytryptamine (5-HT) reuptake inhibitors and melatonin receptor agonists are often used for treatment, such as Psilocybin, fluoxetine, paroxetine, agomelatine, and so on. However, they have a slow onset, low remission rate, and different degrees of side effects such as dry mouth, blurred vision and memory loss, anxiety, transient headaches, and so on (Carhart-Harris et al., 2016). Traditional Chinese Medicine (TCM), based on the advantages of multiple targets, multiple approaches, and multiple levels, has strengthened depression treatment to a large extent (Chi et al., 2019).

Epigenetics is mostly studied in the case of constant gene nucleotide sequences. The expression of genes is mostly caused by factors such as the environment, emotion, and disease. Therefore, the variation caused by epigenetics is mostly caused by changes in environmental factors and nucleotide interactions. Epigenetic modification, such as histone modification, DNA methylation, and non-coding RNA, affects gene expression. It is generally believed that patients with depression are mostly caused by physiological, psychological, social stress, genetic, and other factors. Similarly, there are differences in epigenetic modification in patients with depression (Nagy et al., 2015), so patients with depression are related to epigenetics.

**FIGURE 1:**
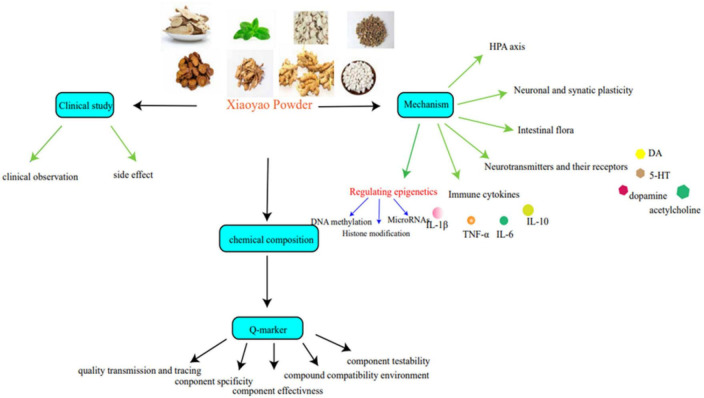
Treatment of depression with Xiaoyao Powder.

Xiaoyao Powder comes from the “Formulary of the Bureau of Taiping People’s Welfare Pharmacy.” It consists of *Bupleurum Chinese* DC. (Radix Bupleuri), *Paeonia lactiflora* Pall. (Paeonia Lactiflora), *Atractylodes macrocephala* Koidz. (Atractylodes macrocephala), *Angelica sinensis (Oliv.)* Diels. (Angelica sinensis), *Poria cocos* (Schw.) Wolf. (Poria cocos), *Glycyrrhiza uralensis* Fisch. (Radix Glycyrrhizae), *Mentha haplocalyx* Briq (Mentha haplocalyx), and *Zingiber officinale* Rosc. (Ginger) (Table 1). It is generally applied in clinical medication to cure anxiety disorder and multiple diseases complicated with depression (Li et al., 2007; Zhang et al., 2022). Compared with western medicine, it can relieve adverse reactions such as bradycardia, lower limb swelling, headache, dry mouth, blurred vision, and so on. Based on systematically collating relevant domestic and foreign research, this review includes an examination of pharmacological action, clinical applications, and the chemical quality markers of Xiaoyao Powder and how it can be used to treat depression to provide quality control references for the subsequent study (Figure 1).

**TABLE 1:** Composition of Xiaoyao Powder.

Botanical name	English name	Part used	Proportion
*Bupleurum Chinese* DC.	Radix Bupleuri	Root	2
*Paeonia lactiflora* Pall.	Paeonia Lactiflora	Root	2
*Atractylodes macrocephala* Koidz.	Atractylodes macrocephala	Rhizome	2
*Angelica sinensis (Oliv.)* Diels.	Angelica sinensis	Root	2
*Poria cocos (Schw.)* Wolf	Poria cocos	Sclerotium	2
*Zingiber officinale* Rosc.	Ginger	Rhizome	2
*Mentha haplocalyx* Briq.	Mentha haplocalyx	The whole grass and leaves	1
*Glycyrrhiza uralensis* Fisch.	Radix Glycyrrhizae	Root and rhizome	1

## Clinical application of Xiaoyao Powder in the treatment of depression

### Clinical observations of Xiaoyao Powder in the treatment of depression

From the Song Dynasty to the present, Xiaoyao Powder was found to have good efficacy in relieving depression. At the moment, Xiaoyao Powder alone or combined with western medicine such as fluoxetine, paroxetine, and so on has quite a good curative effect on depression. The Xiaoyao Powder has been recognized as an effective medicine for depression, and can relieve depressive symptoms with fewer side effects. A study reported on the use of Xiaoyao Powder in mild depression (liver depression and spleen deficiency type in Chinese medicine). The results made clear that Xiaoyao Powder might enhance the clinical therapeutic effectiveness of the treatment group (96.7%) and reduce the HAMD score of patients in the treatment group (Wang, 2020). A study by Chen included 30 patients with depression and treated them with Jiawei Xiaoyao Powder for 8 weeks, and the Hamilton index indicated that Jiawei Xiaoyao Powder had more significant efficacy compared with fluoxetine treatment (Chen and Wu, 2016). Yuan randomly divided 72 depression patients into an experimental group who took Xiaoyao Powder orally combined with Fluoxetine and a control group (oral Fluoxetine), with 36 cases in each group. After 4 weeks of treatment, they found that the experimental group had a better curative effect according to the Hamilton Depression Scale and TCM syndrome score (Yuan et al., 2022). Chen chose 141 depression patients treated with Jiawei Xiaoyao Powder (Xiaoyao Powder plus *Paeonia suffruticosa* Andrews, *Gardenia jasminoides* J. Ellis) and placebo, respectively. The GI symptom score (GIS), Hamilton Depression Scale, and Hamilton Anxiety Scale were observed. The GIS was much higher for those treated with Jiawei Xiaoyao Powder than for those treated with the placebo (Chen et al., 2020). It has also been reported that Xiaoyao Powder combined with other therapies has good curative effects on depression with gynecological diseases, such as breast cancer, perimenopause, and so on (Sun et al., 2016; Tang et al., 2019; Xiao, 2021). Gao (2017) found that 70 patients with postpartum depression were selected and treated with Xiaoyao Powder and paroxetine hydrochloride, respectively. After 12 weeks of treatment, they found that the total effective rates of Xiaoyao Powder and paroxetine hydrochloride were 97.14 and 77.14%, respectively. The incidence of adverse reactions was 2.86 and 25.71%, suggesting that Xiaoyao Powder can significantly improve the symptoms of patients with depression, and high safety. Zheng et al. (2021) randomly divided 150 patients with anxiety and depression into an observation group and a control group who took Xiaoyao Powder, Flupentixol, and Melitracen Tablets, respectively. After 4 weeks of treatment, HAMD (86.3%) and HAMA (79.4%) were higher in the observation group (77.8%) than HAMA (72.2%) in the control group. The anxiety/somatization factor score of the HAMD scale in the observation group (3.5 ± 1.4) was significantly lower than that in the control group (4.6 ± 1.3), and the sleep disorder factor score (2.6 ± 1.3) was significantly lower than that in the control group (3.3 ± 1.4). This shows that Xiaoyao Powder and Flupentixol and Melitracen tablets have obvious effects on improving the symptoms of mixed anxiety and depression, but that Xiaoyao Powder is superior to Flupentixol and Melitracen tablets in improving anxiety, somatization, and sleep disorders and long-term effects. Jiao J. et al. (2019) selected 48 patients with depression syndrome of liver depression and spleen deficiency after chemotherapy for breast cancer, and randomly divided them into a control group (Lifumin tablets) and a traditional Chinese medicine group (Xiaoyao Powder group). After 8 weeks of continuous treatment, the TCM syndrome score of the Xiaoyao Powder group decreased by 12.96 points after treatment, and the total effective rate of treatment was 87.5%. The TCM syndrome score of the control group decreased by 4.96 points, and the total effective rate of treatment was 45.8%. Therefore, Xiaoyao Powder can effectively alleviate the depression syndrome of liver depression and spleen deficiency type in patients with breast cancer undergoing post-operative chemotherapy, and can significantly improve the TCM syndrome score, and the treatment is safer and more effective, with better clinical application value.

### Xiaoyao Powder can improve adverse reactions to depression

The drugs for depression currently include fluoxetine, Paxil, propranolol, venlafaxine, and flecainide. However, through clinical observations, patients often present with bradycardia, lower limb swelling, headache, and other clinical manifestations (Woron et al., 2019). A clinical investigation randomly divided 78 patients into an experimental and control group. The patients in the control group were treated with fluoxetine hydrochloride dispersive tablets and the other group was used to cure fluoxetine hydrochloride dispersive tablets combined with modified Xiaoyao Powder. It was found that the Hamilton Depression Scale was lower in the experimental group (*P* < 0.05). The experimental group displayed fewer adverse reactions such as dizziness, insomnia, gastrointestinal discomfort, dry mouth, headache, and constipation (Lv, 2015). In another study, 80 patients were assigned to two groups (Xiaoyao Powder and paroxetine separately). Compared with the western medicine group, the effective rate of the TCM group was significantly higher. The occurrence of adverse reactions such as headache, constipation, and rapid heartbeat was reduced for the 2 months of treatment. There were fewer adverse reactions in the TCM group. The effectiveness rate was 95% higher than that of the western drug group (77.5%) (Ma et al., 2020). In addition, Zheng (2021) found that Xiaoyao Powder combined with sertraline on post-stroke depression might enhance the efficacy of sertraline and reduce adverse reactions, which is worth promoting (Table 2).

**TABLE 2:** Xiaoyao powder improves adverse reactions to depression.

Number	Group	Medication	Number of people	Age	Adverse effect	The medication time
1	Observation group	Bupleurum Chinese DC. Paeonia lactiflora Pall. Atractylodes macrocephala Koidz. Angelica sinensis (Oliv.) Diels. Poria cocos (Schw.) Wolf Glycyrrhiza uralensis Fisch. and fluoxetine	39	27–64	Gastrointestinal discomfort: 1 Lose sleep: 1 Dizzy: 1 Dry: 0 Astriction: 0 Headache: 0 Total incidence: 7.69%	From January 2014 to January 2015
	Control group	Fluoxetine	39	26–61	Gastrointestinal discomfort: 2 Lose sleep: 1 Dizzy: 2 Dry: 2 Astriction: 2 Headache: 1 Total incidence: 25.64%	From January 2014 to January 2015
2	Observation group	Bupleurum Chinese DC. Paeonia lactiflora Pall. Atractylodes macrocephala Koidz. Angelica sinensis (Oliv.) Diels. Poria cocos (Schw.) Wolf Glycyrrhiza uralensis Fisch. and paroxetine tablets	40	20–54	Vomit: 6 Headache: 3 Astriction: 1 The heart beats faster: 2 Total incidence: 10.00%	From March 2017 to August 2019
	Control group	Paroxetine tablets	40	20–54	Vomit: 2 Headache: 1 Drowsiness: 1 Total incidence: 30.00%	From March 2017 to August 2019
3	Observation group	Bupleurum Chinese DC. Paeonia lactiflora Pall. Atractylodes macrocephala Koidz. Angelica sinensis (Oliv.) Diels. Poria cocos (Schw.) Wolf Glycyrrhiza uralensis Fisch. and sertraline citrate tablets	25	30–66	Diarrhea: 0 Lose sleep: 1 Astriction: 0 Feeble: 1 Total incidence: 8%	From May 2019 to May 2020
	Control group	Sertraline citrate tablets	25	31–69	Diarrhea: 1 Lose sleep: 2 Astriction: 1 Feeble: 2 Total incidence: 24%	From May 2019 to May 2020

## Pharmacological mechanisms of Xiaoyao Powder in the treatment of depression

### Xiaoyao Powder adjusts the HPA axis

Modern research has shown that the incidence of depression has a bearing on the dysfunction of the HPA axis (Cai et al., 2015; Zhou et al., 2022). In addition, the HPA axis of the hypothalamus can promote the expression of corticotropin-releasing factor (CRF), which is transferred from the portal system to the pituitary gland to synthesize ACTH and stimulate the production of cortisol in the adrenocortical cortex (Olloquequi et al., 2018). Wu found that Jiawei Xiaoyao Powder could relieve depression-like behavior and hyperactivity of the HPA axis (Wu et al., 2016). O’Carroll et al. (2003) found that chronic stress reduced the expression of apelin in the PVN and the content of cortisol in plasma, and increased the expression of APJ, meaning it has a negative feedback effect with the HPA axis. Xiaoyao Powder can inhibit the changes in the apelin/APJ system in depressed mice by upgrading the apelin level and downgrading the APJ level (Yan et al., 2018).

### Xiaoyao Powder regulates neurotransmitters and their receptors

The neurotransmitter hypothesis is one of the widely accepted mechanisms of gloomy, which consists of the monoamine hypothesis and glutamate-mediated excitatory toxicity hypothesis. The HPA axis is controlled by monoamine neurotransmitters such as 5-HT to relieve emotional stress (Goel et al., 2011). Zhang et al. (2018) found that the addition of the traditional medicine Xiaoyao Powder increased the expression of cortisol and tyrosine hydroxylase, reduced the levels of monoamine neurotransmitters (5-HT and norepinephrine), and alleviated the depressive phenotype and depressive-like behavior of zebrafish. Other scholars have found that Xiaoyao Powder can expand the level of 5-HT in the central nervous system of depression rats and reduce the content of 5-HIAA in depression rats, thus improving depressive symptoms (Bao et al., 2008). Tryptophan metabolism plays a key role in mental diseases. Xiaoyao Powder can up-regulate the IDO1, down-regulate TPH2 to improve tryptophan metabolism, and increase the contents of canine urine, 5-hydroxytryptophan to play an antidepressant role (Jiao H. Y. et al., 2019). Ding found that Xiaoyao Powder could inhibit the release of NE into the blood by LC-NE neurons, resulting in increasing NE in rats, thus playing an antidepressant role (Ding et al., 2014). Wang argued that Xiaoyao Powder shortened the recovery time of post-stroke depressed rats, and regulated the mRNA expression of BDNF. The CB1R and CB2R were upgraded, while the expression of CRF was downgraded (Wang et al., 2019). Glutamic acid is the most abundant amino acid, which is the main excitatory neurotransmitter (Brosnan and Brosnan, 2013). Li (2020) found a strategy that can quicken the effects of antidepressants. For example, Rapid E/I balance is achieved by relieving the inhibition of GABA interneurons on glutamatergic pyramidal neurons and directly activating pyramidal neurons. 5-HT transporters bind to some receptors such as 5-HT1A/1B, and regulate 5-HT neuronal activity and Glu/GABA balance etc (Li, 2020). Luo et al. (2013) found that the biochemical effects of Xiaoyao Powder were characterized by elevated levels of glutamic acid in plasma.

### Xiaoyao Powder regulates synaptic plasticity

Synaptic plasticity is the basis for the stability of studying and memory function and plays a regulatory part in sleep, anxiety, and depression (Lovinger and Abrahao, 2018). In the study of depression, many scholars have found that there is a correlation between synaptic plasticity and depression (Alves et al., 2017). Li X. H. et al. (2017) found that Xiaoyao Powder could reduce the damage caused by stress on original synapses, accelerate the connection and formation of new synapses, and thus maintain synaptic plasticity. In most patients with depression, the synaptic structure and synaptic plasticity that are changed are universal. From the perspective of the mechanism, BDNF is essential for the growth, proliferation, survival, and synaptic activity of neurons, playing a decisive part in the survival of neurons and synaptic activity (Machaalani and Chen, 2018; Chen et al., 2022). The BDNF is critically decreased in mood disorders and plays an essential role in most anti-depressant treatments (Caviedes et al., 2017). Ding et al. (2017) found that Xiaoyao Powder could also significantly improve the body weight and depression-like behavior of CUMS mice, significantly increase the BDNF and GDNF in serum, and add the standard of GFAP protein, mRNA as well as immune reactivity in the hippocampus.

### Xiaoyao Powder regulates intestinal flora disorders

All kinds of chronic stress can lead to the imbalance of intestinal flora in the body, causing inflammation in the intestinal tract and affecting the absorption of nutrients by intestinal flora. Some surveys have found that severe depression was associated with microbial imbalance. An AI-2 molecule was produced by segmental filamentous bacteria in the intestinal flora. The AI-2 molecule was found to reduce the expression of serum amyloid proteins SAA1 and SAA2, and changed the distribution of inflammatory factors in the brain through the interaction between inflammatory factors and the brain (Medina-Rodriguez et al., 2020). Zhu et al. (2019) believed that Xiaoyao Powder could regulate the balance of intestinal flora, enhance the abundance of Firmicutes, Bacteroidetes, Proteobacteria, and Ruminococcus, reduce the abundance of Prevotellaceae_Ga6A1_group, Prevotellaceae_UCG-001, Desulfovibrio and regulate its metabolites such as short-chain fatty acids so as to play an antidepressant role. Other studies have indicated that Xiaoyao Powder can regulate the excessive abundance of Proteobacteria, Firmicutes, and Cyanobacteria in rats with chronic and unpredictable stress, which restores them to normal levels, thus achieving the effect of treating functional dyspepsia and LSSD after exposure to CUMS (Qiu et al., 2017).

### Xiaoyao Powder regulates inflammation cytokines

At present, it is found that cellular inflammatory factors can promote the incidence of depression, and present hypercortisolemia by directly activating the abnormal regulation of the hypothalamus-pituitary-adrenal axis and indirectly changing the sensitivity of glucocorticoid receptors to cortisol, resulting in hypercortisolemia. Cytokines consume central synaptic 5-HT levels by reducing synaptic 5-HT synthesis and increasing its reuptake. They may also deplete neurotrophic factors (Makhija and Karunakaran, 2013). Studies have shown that depressive patients demonstrate increased secretion of pro-inflammatory cytokines including interleukin-1β (IL-1β), interleukin-6 (IL-6), and tumor necrosis factor (TNF-α), cut down the amounts of anti-inflammatory cytokines such as interleukin-4 (IL-4) and interleukin-10 (IL-10). Experimental studies have shown that Xiaoyao Powder can effectively reduce the expression of IL-6 and TNF-α in the hippocampus of rats induced by chronic and unpredictable mild stress (Wan et al., 2020). Jiawei Xiaoyao Powder can elevate the depression-like behavior of LPS-induced rat models with depression, downregulating the overexpression of IL-1β, IL-6, and TNF-α mRNA in the hippocampus (Yu et al., 2017). Xiaoyao Powder can decrease the LPS in the stool, blood, and colon tissue and reduce inflammatory cytokines such as IL-1β, NLRP3, ASC mRNA, and DNA in the colon, thus relieving depression and other symptoms (Hao et al., 2021). It can regulate the hyperfunction of the HPA axis, the immune balance of Th2 and Th17 cells in rats under chronic bondage stress, and reduce the plasma ACTH, serum CORT, IL-13, and IL-17 (Li X. H. et al., 2017).

## Chinese medicine in the treatment of depression based on epigenetics

### The relationship between epigenetics and depression

DNA methylation is a natural modification. In mammals, DNA methylation is an epigenetic mechanism, that mainly refers to the methyl transfer to the C5 position (Moore et al., 2013). Studies have found that some diseases of external environmental stress like post-traumatic stress disorder are closely related to DNA methylation (Klengel et al., 2014). At present, relevant scholars have found that the abnormal expression of RUFY3 and GBBR2 genes in the brain of patients with depression is increased, and the expression of GRIK2 and BEGAIN is decreased. The abnormal expression of BDNF, SLC6A4, ZBTB20, HDAC2, and HDAC5 genes in the surrounding tissues increased, and the expression of GRIN2A and WDR26 decreased (Saavedra et al., 2016; Penner-Goeke and Binder, 2019). Histone modification is mostly caused by chromatin remodeling, mainly nucleosomes, histones, and corresponding DNA molecules change. The common modification methods mainly include acetylation, methylation, ubiquitination, etc (Li et al., 2008), of which acetylation is the most common modification form. This process is regulated by histone deacetylase (HDAC) and histone acetyltransferase (HAT) to regulate gene activation by regulating the acetylation of histones or transcription factors. Scholars believe that histone acetylation-related genes can be used as potential biomarkers of peripheral blood vessels in patients with depression, which can inhibit HDAC or improve depression-like behavior with antidepressant combination therapy (Schroeder et al., 2007; Hobara et al., 2010). MicroRNAs are endogenous non-coding RNAs with regulatory functions in eukaryotes. They can bind to miRNA response elements (MREs) in the 3′-untranslated regions (3′UTRs) of target genes through sequence complementation to degrade or inhibit the translation of target genes, thereby inhibiting the level of target genes. A variety of studies have shown that miRNAs play a key role in the pathogenesis of neurological diseases such as depression (Lopez et al., 2014; Dwivedi, 2018; Muñoz-Llanos et al., 2018).

### Traditional Chinese medicine can treat depression through epigenetics

DNMT3L is an important regulator of DNA methylation (Wienholz et al., 2010). The change of the MBP gene may be one of the pathological changes of depression. Li (2011) induced depression rat model by CUMS. Acupuncture at “Baihui” and “Yanglingquan” can increase the mRNA expression level of the DNMT3L gene and decrease the mRNA expression level of the MBP gene in a depression rat model. Studies have shown that the gene expression of BDNF is closely related to the expression of methyl CpG binding protein 2 (MeCP2), a key molecule involved in epigenetic modification (Jiang, 2008), and MeCP2 can inhibit DNA methylation, chromatin remodeling, and transcription (Yu et al., 2006c). Shi et al. (2018) found that proanthocyanidins can increase the level of MeCP2, carry out epigenetic modification, and then increase the protein expression of depression-related molecule BDNF, thereby improving the cognitive function of depression. Yin et al. (2017) found kidney-tonifying and qi-invigorating prescriptions, such as Zuogui Pill and Yiqi Congming Decoction. It can up-regulate the expression of the egr1 gene and reduce the methylation rate of the egr1 region, increasing the learning and memory ability of elderly rats, and improving cognitive emotion. Ren et al. (2012) found that compared with untrained women, the DNA methylation status of 6 CpG sites that should increase with age was significantly lower than normal. Related studies have found that depressed animals can inhibit the role of histone deacetylases (HDACs) in the brain and inhibit HDAC in the nucleus accumbens, hippocampus, and amygdala (Covington et al., 2015). Covington et al. (2011) found that the level of acetylated histone H3 (acH3) in the amygdala and hippocampus of mice with chronic social stress failure continued to decrease. Traditional Chinese Medicine has various forms of antidepressant effects through histone modification. For example, ginsenosides Rg1 and Rb1 in ginseng can increase the phosphorylation of amygdala protein kinase (APKA) and cyclic adenosine monophosphate effect element binding protein, activate the cyclic adenosine monophosphate-cyclic adenosine monophosphate effect element binding protein-brain-derived neurotrophic factor (cAMP-CREB-BDNF) circuit in the prefrontal cortex, improve synaptic structural abnormalities in the prefrontal cortex, and thus improve depression (Liu et al., 2016). Su et al. (2014) found that Xiaochaihu Decoction can increase the expression of 5-hydroxytryptamine, BDNF, nerve growth factor, and TrkB mRNA in the hippocampus of the model. In addition, acupuncture can improve the depression-like behavior of CUMS model rats by increasing the phosphorylation level of ERK1/2 and increasing the expression of neurotrophic factor BDNF protein, thereby alleviating depressive symptoms (Li et al., 2018). Jiang et al. (2018) found that acupuncture could down-regulate the expression of HDAC2 protein in the hippocampus, promote the acetylation of histone H3K9 in the hippocampus, increase the expression of BDNF mRNA, and alleviate depression-like behavior. MiRNA is the most studied and characterized, and has become the main regulator of neural plasticity and advanced brain function. Yang et al. (2015) found that exosomes secreted by mesenchymal stem cells after intervention with Buyang Huanwu decoction can increase the expression of miR-126 and decrease the expression of miR-221 and miR-222, thereby improving cognitive function. Lily Rehmannia decoction can change a series of miRNAs in depression. For example, miR-144-3p down-regulates targeting glutamate decarboxylase 1 (Gad1) and anti-vesicular GABA transporter (VGAT), and down-regulates miR-495-mediated BDNF expression to exert antidepressant effects (Zhang H. et al., 2021). Duan et al. (2016) found that the expression of miR-383-5p and miR-764-5p was down-regulated in CUMS rats after electroacupuncture intervention, suggesting that electroacupuncture may play an antidepressant role by promoting neurotrophic and inhibiting neuronal apoptosis signaling pathways.

### Xiaoyao Powder can treat depression through epigenetics

Li (2018) found that Xiaoyao Powder can effectively affect the insulin signaling pathway by up-regulating the candidate target gene Slc2a4 regulated by differential methylation in the hypothalamic arcuate nucleus of depression model rats and down-regulating Nmur2, thereby improving depression. Through Sequenom Mass ARRAY, Sun et al. (2018a,b) found that the methylation of some specific regions in Npas2 and Stk32 increased after chronic stress. After taking Xiaoyao Powder, the expression of Npas2 m RNA and its corresponding protein decreased, and the degree of DNA methylation at the corresponding sites decreased significantly (Sun et al., 2018a,b).

## Study on the chemical constituents of Xiaoyao Powder

### The chemical constituents of Xiaoyao Powder

The chemical components of *Bupleurum Chinese* DC. Mainly include saponins, volatile oils, flavonoids, and so on. Studies have found that citric acid, laurene, ethanoic acid, and other components are present in the flowers, leaves, stems, roots, and fruits of *Bupleurum Chinese* DC., as determined by GC-MS (Meng et al., 2014). Fifty compounds such as saikosaponin A, saikosaponin D, saikosaponin B3, and saikosaponin B1 were detected in *Bupleurum Chinese* DC. by means of the UHPLC-Q-TOF-MS technique (Lei et al., 2018). Liu et al. (2017) determined saikosaponin B1, saikosaponin F and saikosaponin W etc., in *Bupleurum Chinese* DC. Root using the spectrometric method. The HPLC method was used to determine the contents of quercetin, isorhamnetin, and kaempferol in *Bupleurum Chinese* DC. The results showed that the contents were as high as 4.7081, 0.7740, and 0.09515 mg g^–1^, respectively (Lin et al., 2012).

The chemical components of *Angelica sinensis (Oliv.)* Diels. mainly include benzene peptides, polysaccharides, and so on. Zhang et al. (2016) discovered a new skeleton of phenyl peptide trimmers and phenyl peptide dimers containing peroxy bridge groups. Later, Two pairs of phthalide trimers such as (−)/(+) triligustilides A (1a/1b) and (−)/(+) triligustilides B (2a/2b) were isolated from *Angelica sinensis (Oliv.)* Diels. (Zou et al., 2018). Zhao et al. (2023) detected twelve chemical components by high-performance liquid chromatography and triple quadrupole mass spectrometry method. Polysaccharides were found to be one of the main active components in *Angelica sinensis (Oliv.)* Diels. (Zhao et al., 2016). Additionally, it was found that polysaccharides from *Angelica sinensis (Oliv.)* Diels. were mainly divided into acid polysaccharides and neutral polysaccharides (Sun et al., 2005).

The chemical components of *Atractylodes macrocephala* Koidz. mainly include lactones, coumarins, polysaccharides, volatile oils, and so on. Li found two new eudesmane-type sesquiterpenes in the rhizome of *Atractylodes macrocephala* Koidz., namely eudesma-4(15),7(11)-dien-8α,12-ether (atractylenother, 1),8α-hydroxyeudesma-4(15),7(11)-dien-8β,12-olide (8-epiatractylenolide III, 2), and one new natural product named 4(R),15-epoxy-8 beta hydroxy-atractylenolide II. In addition, it was found that coumarins and phenylpropyl compounds isolated from the rhizomes and aboveground parts of *Atractylodes macrocephala* Koidz. in recent years were mainly daphne, artemisinin, scopolamine, umbelactone, and so on (Li and Yang, 2014). Wang et al. (2014) detected atractylodes’ polysaccharides, mainly including rhamnose-xylose, mannose, and galactose by high-performance gel permeation chromatography and gas chromatography-mass spectrometry. By using GC-TOF-MS analysis, they found that 26 compounds were reduced in stir-fried bran as compared to raw bran. It indicated that the processing method had a significant influence on the types of volatile components in *Atractylodes macrocephala* Koidz (Zhang et al., 2014).

The chief chemical constituents of *Paeonia lactiflora* Pall. are terpenoids, polyphenols, flavonoids, and so on. Studies have found that triterpenoids such as oleanolic acid, ivy saponin, paeoniflorin, betulinic acid, 11α,12α-epoxy-3β, 23-dihydroxy-30-noroleanol-20-ene-28, and 13β-cycloester are the main constituents of *Paeonia lactiflora* Pall. (Zhou and Wang, 2017). Flavonoids in *Paeonia lactiflora* Pall. are mainly compounds with a 2-phenylchromogen structure, including catechin, 4′,5-dihydroxy-flavone-7-o-β-D-glucoside, and 5,7-dihydroxy-flavone-4′-o-β-d-glucoside (Shu et al., 2014).

The major chemical components of *Poria cocos (Schw.)* Wolf are polysaccharides, triterpenoids, sterols, and others. In recent years, scholars have isolated and purified PCSG, PCS3-II, PCM3-II, galactose, glucose, mannose, and other polysaccharide compounds (Shen et al., 2012). Yang et al. (2014) obtained triterpenoids such as oleanolic acid, oleanolic acetate, and alpha-aromatic resin alcohol acetate through their experiments by mass spectrometry and nuclear magnetic resonance. Wang et al. (2015) identified the components in *Poria cocos (Schw.)* Wolf Skin and White *Poria cocos (Schw.)* Wolf Skin by UHPLC-DAD-FT/MS. They discovered that dehydrotumulosic acid, trametenolic acid, dehydrotrametenolic acid, and poricoic acid A were the main compounds.

The main chemical components of Processed *Glycyrrhiza uralensis* Fisch. are flavonoids, coumarin, and other components. Processed *Glycyrrhiza uralensis* Fisch. mainly contains flavonols, isoflavones, chalcone, and other flavonoids (Li et al., 2000). Through UPLC-Q-TOF-MS Cui found that the contents of five flavonoids such as glycyrrhiza flavonol, glycyrrhiza A, isoflavone and so on increased, while the contents of two coumarins such as 7,2′,4′-trihydroxy-5-methoxy-3-aromatic coumarin and Hedysarimcoumestane B astragalus coumarin B decreased significantly after the processing of *Glycyrrhiza uralensis* Fisch. It indicates that the composition of *Glycyrrhiza uralensis* Fisch. changes after processing (Cui et al., 2020).

The other type of herbs are *Zingiber officinale* Rosc. and *Mentha haplocalyx* Briq. They mainly have contains volatile oil components. The researchers found that by gas chromatography-mass spectrometry, the *Zingiber officinale* Rosc. associated irritants include zingerone, shogaols, gingerols, paradols, wikstromol, and so on (Idris et al., 2019). And someone has detected that the piperitenone oxide and carvone are rich in content in the *Mentha haplocalyx* Briq (Kowalczyk et al., 2022).

### Analysis of quality markers of Xiaoyao Powder in the treatment of depression

Some studies have explored the components of Xiaoyao Powder, but few studies have examined its quality markers. Quality markers are a new concept proposed by Changxiao Liu (Li Y. B. et al., 2019), which mainly include “quality transmission and tracing,” “component specificity,” “component effectiveness,” “compound compatibility environment,” and “component testability.” The components of traditional Chinese medicine and compound are complex and diverse, and there are many targets for the treatment of diseases. The quality of single traditional Chinese medicine will affect the efficacy of the compound, so it is necessary to establish a complete quality evaluation system to control the traditional Chinese medicine compound. Therefore, the article is determined by the quality markers of Xiaoyao Powder in the treatment of depression, which has more clinical application value and the feasibility of establishing a quality control system throughout the whole process.

### Q-marker prediction based on mass transfer and traceability

*Bupleurum Chinese* DC., *Angelica sinensis (Oliv.)* Diels., *Atractylodes macrocephala* Koidz., *Paeonia lactiflora* Pall., *Poria cocos (Schw.)* Wolf., Processed *Glycyrrhiza uralensis* Fisch., *Zingiber officinale* Rosc. and *Mentha haplocalyx* Briq. are the key ingredients in Xiaoyao Powder, and these were used as keywords in Q-marker prediction. A total of 1,352 chemical components were retrieved from the TCSMP database,^1^ including 349 came from *Bupleurum Chinese* DC., 120 for *Angelica sinensis (Oliv.)* Diels., 55 for *Atractylodes macrocephala* Koidz., 85 for *Paeonia lactiflora* Pall., 34 for *Poria cocos (Schw.)* Wolf, 220 for Processed *Glycyrrhiza uralensis* Fisch., 164 for *Mentha haplocalyx* Briq. and 265 for *Zingiber officinale* Rosc. According to oral drug bioavailability (OB) ≥30% and drug-like (DL) ≥0.12, the following active ingredients were screened out: 14 for *Bupleurum Chinese* DC. (saikosaponin a, quercetin, kaempferol, etc.), 4 for, *Angelica sinensis (Oliv.)* Diels. (rohamberyl, β-sitosterol, stigmasterol, etc.), 12 for *Atractylodes macrocephala* Koidz. (balanol, atractylodes I, atractylodes II, etc.), 13 for *Paeonia lactiflora* Pall. (methyl trans linoleate, oleanolic acid, paeoniflorin, etc.), 13 for *Poria cocos (Schw.)* Wolf. (poria acid, dehydroporia acid, trametenolic acid, etc.), 94 for Processed *Glycyrrhiza uralensis* Fisch. (kaempferol, glycyrrhiza A, urchin etc.), 16 for *Zingiber officinale* Rosc. (6-gingerol, 6-shogaol, beta-sitosterol, etc.), and 13 for *Mentha haplocalyx* Briq. (fortunellin, acacetin, linarin, etc.).

The effective components of the compound enter the blood, reach the relevant targets, and exert their activity, which are the main features of the efficacy of the compound prescription. Xu et al. (2018) found that quercetin, ferulic acid, and liquiritigenin in Xiaoyao Powder entered the blood faster, respectively (Tmax = 0.10 ± 0.03,0.21 ± 0.10,0.19 ± 0.07) through UPLC-MS. The absorption of atractylenolide II and atractylenolide III into blood was slow (Tmax = 0.64 ± 0.29,0.67 ± 0.26). Saikosaponin a, saikosaponin c and glycyrrhizic acid were absorbed into the blood due to factors such as enterohepatic recirculation and changes in gastric emptying, and the blood concentration-time curve showed a bimodal type (Xu et al., 2018). Other scholars found that gamma-octalactone, paeoniflorin, and p-hydroxybenzoic acid were the main blood components of *Bupleurum Chinese* DC. and *Paeonia lactiflora* Pall. through UPLC-Q-TOF-MS (Zhang et al., 2019). Through UPLC-Q-Orbitrap-HRMS technology, L-tryptophan and atractyloside A were found to be the main blood components of *Atractylodes macrocephala* Koidz. (Zheng et al., 2022). Lu discovered that Senkyunolide I, senkyunolide H, etc., are considered as an entry component of *Angelica sinensis (Oliv.)* Diels. (Lu et al., 2017). In addition, through the analysis of UPLC-TOF-MS, Neng found that the main prototype components of *Poria cocos (Schw.)* Wolf. in the blood were poria C, dehydrosleumoic acid, and oleanolic acid (Neng et al., 2020). Li L. L. et al. (2019) analyzed eight components of *Zingiber officinale* Rosc. into blood such as 6-gingerol, 6-shogaol, 8-gingerol and so on. Therefore, gamma-octalactone, paeoniflorin, p-hydroxybenzoic acid, L-tryptophan, atractyloside A, polylactic acid C, dehydrostrymoic acid, oleanolic acid, and gingerol may be the direct active components of Xiaoyao Powder.

### Prediction of Q-markers based on component specificity

Radix Bupleuri is a plant in the umbrella family *Bupleurum Chinese* DC. and *Bupleurum scorzonerifolium* Willd. The dried roots of this plant mainly contain saponins, sterols, volatile oils, polysaccharides, and other components. It is generally believed that saponins and sterols are the material basis of Radix Bupleuri. Therefore, saikosaponin can be used as the unique component of Radix Bupleuri (Sui et al., 2021).

Angelica sinensis is the dried root of *Angelica sinensis* (Oliv.) Diels. It mainly includes volatile oils, vitamins, organic acids, and other components. It is generally believed that volatile oils and organic acids are the material basis of Angelica sinensis. Therefore, angelic lactone and ferulic acid can be used as the specific components of angelica sinensis (Li et al., 2022).

Atractylodes macrocephala is the dried rhizomes of *Atractylodes macrocephala* Koidz. The dried rhizomes of this plant mainly contain volatile oils, flavonoids, glycosides, and other components. It is generally believed that volatile oils and flavonoids are the material basis of Atractylodes macrocephala. Therefore, atractylodes ketone, atractylodes lactone can be used as the specific components of Atractylodes macrocephala (Wang et al., 2022).

Paeonia Lactiflora is the dried root of *Paeonia lactiflora* Pall. It mainly contains volatile oil, glycosides, resins, tannins, and other components. It is generally believed that glycosides and volatile oils are the material basis of Paeonia Lactiflora. Therefore, paeoniflorin and methyl antilinoleate can be used as the specific components of Paeoniae Lactiflora (Tanaka et al., 2013).

Poria cocos is the dried sclerotium of *Poria cocos* (Schw.) Wolf. It mainly contains polysaccharides, organic acids, amino acids, and other components. It is generally believed that polysaccharide and organic acid are the material basis of Poria cocos. Therefore, the poria polysaccharides, dehydromomoic acid, and oleanolic acid can be used as the specific components of Poria cocos (Lu et al., 2022).

Radix glycyrrhizae is the dried roots and rhizomes of *Glycyrrhiza uralensis* Fisch, *Glycyrrhiza inflata* Bat, and *Glycyrrhiza glabra* L. It mainly contains polysaccharides, flavonoids, organic acids, and other components. It is generally believed that polysaccharides and organic acids are the material basis of Radix glycyrrhizae. Therefore, the glycyrrhiza polysaccharides, glycyrrhiza glycoside, and glycyrrhizic acid can be used as the specific components of Radix glycyrrhizae (Lu et al., 2022).

Ginger is a fresh rhizome of *Zingiber officinale* Rosc. It mainly includes volatile oils. It is believed that it is the effective material base of Ginger, and gingerol is a specific component of Ginger (Li et al., 2013).

Mentha haplocalyx is the whole grass and leaves of the *Mentha haplocalyx* Briq. It also mainly has volatile oils. Some think that it is the effective material base of Mentha haplocalyx or menthol (Zhang et al., 2015).

## Quality markers of Xiaoyao Powder in the treatment of depression based on the correlation between components and pharmacodynamics

### *Bupleurum Chinese* DC.

The main antidepressant active ingredients of Xiaoyao Powder are paeoniflorin, quercetin, saikosaponin D, and so on. Zhang S. et al. (2021) found that Kaempferol can increase the levels of phosphorylated (p) PI3K/PI3K and pAKT/AKT in the hippocampus of CUMS mice, and reduce pGSK3 β/GSK3 β level. Studies have shown that quercetin in bupleurum can significantly reverse anxiety and depression-like behavior induced by corticosterone-releasing factor (CRF) in rats (Bhutada et al., 2010). It was also detected that quercetin could notably inhibit the expression of CRF mRNA, thus achieving the inhibition of hypothalamic–pituitary–adrenal axis hyperfunction (Kawabata et al., 2010). Dimpfel confirmed that quercetin can significantly change the frequency of brain waves and inhibit the activity of monoamine oxidase in rats (Dimpeel, 2009). Saikosaponin D can protect PC12 cells from corticosterone-induced damage by regulating mitochondrial and nuclear corticosteroid receptor transport, partially reversing mitochondrial dysfunction, and inhibiting the mitochondrial apoptosis pathway (Li et al., 2014). Saikosaponin A significantly inhibited MAPK and NF-κβ signaling pathways (Zhu et al., 2013).

### *Paeonia lactiflora* Pall.

Sun found that paeoniflorin in Peony has a significant protective effect on PC12 cells by significantly inhibiting intracellular calcium overload, stabilizing mitochondrial membrane potential, and inhibiting Bax expression, thus promoting Bcl-2 expression and inhibiting cell apoptosis (Sun et al., 2012). Cui found that paeoniflorin in Peony can play an antidepressant role by enhancing the serotonin and monoamine neurotransmitter content (Cui and Jin, 2012). Li et al. thought that paeoniflorin in Paeoniae Radix Alba reduced IFN-α-induced inflammation in serum and brain regions, such as IL-2, IL-4, etc. The depressive-like behavior of mice induced by long-term high-dose IFN-α was also improved (Li J. et al., 2017).

### *Angelica sinensis* (Oliv.) Diels

Angelica ferulic acid can inhibit glutamate uptake to treat mental illness (Yu et al., 2006a). Senkyunolide can protect PC12 cells which can be damaged by glutamic acid and corticosterone. Thus, Senkyunolide may treat psychiatric disorders (Gong, 2019). Zhang et al. (2007) used a forced swimming test, tail suspension test, and drug interaction model in rats and mice to explore the antidepressant mechanism of ferulic acid. They found that sodium ferulate can significantly shorten the swimming time of rats and mice and the immobility time of tail suspension in mice, suggesting that sodium ferulate has an antidepressant effect. By giving excessive Glu or monosodium glutamate to mice, it can cause neuronal deformation in mice and affect the learning and memory ability of mice, while sodium ferulate can improve this situation, suggesting that sodium ferulate exerts antidepressant effects through neuroprotection and neurorestoration pathways (Yu et al., 2006b; Zhang et al., 2008).

### *Zingiber officinale* Rosc.

Curcumin in ginger can significantly increase the number of head twitches in mice caused by 5-HT, antagonize the hypothermia of mice caused by high-dose apomorphine, and increase the amount of 5-HT, NA, dopamine (DA) in the brain, thus playing an antidepressant role (Xu et al., 2005). Kulkarni et al. (2008) further found that Curcumin not only significantly increased the levels of 5-HT and NA in the hippocampus and frontal cortex, but also significantly increased the levels of DA in Striatum and frontal cortex. Chen et al. (2008) also used this model to conduct experiments and found that in the model group, the adrenal/body mass ratio, Adrenal cortex density, serum Corticosterone level, and Glucocorticoid receptor (GR) mRNA expression were increased, and Curcumin could reverse these changes, and also reverse the reduction of Brain-derived neurotrophic factor (BDNF) in rats caused by stress.

## Others

Chen et al. (2021) believed that pachymaran in Poria cocos could increase the levels of BDNF, 5-HT, DA, NE, and other Neurotrophins in the hippocampus of rats, significantly reduce the level of Glu, and thus play an antidepressant role. Glycyrrhizin in licorice can significantly reverse the behavioral damage of rats caused by chronic stress and may achieve an antidepressant effect by improving the activity of Superoxide dismutase, eliminating free radicals, preventing lipid peroxidation, and reducing the production of Malondialdehyde (Zhao et al., 2006). Atractylenolide III can inhibit the increase of pro-inflammatory factors such as IL-1, IL-6, and TNF-α, and alleviate CUMS-induced depression and anxiety-like behavior in rats (Zhou et al., 2021). Gao et al. (2018) showed that atractylenolide I reduced the production of IL-1βby inhibiting the activation of NLRP3 inflammasome, and played an antidepressant-like role in a mouse model of depression induced by chronic unpredictable mild stress (CUMS).

In conclusion, quercetin, saikosaponin D, paeonin, and ferulic acid may be the main ingredients of Xiaoyao Powder in the treatment of depression. They can be used as an important reference for the quality markers of it.

### Quality markers in the treatment of depression based on network pharmacology combined with the environment

The main clinical application of TCM is in compounds. The same traditional Chinese medicine has different effects and effective material bases in different traditional Chinese medicine compounds. It is necessary to predict the quality markers of the unique characteristics of Xiaoyao Powder in the treatment of depression based on network pharmacology in the compatibility environment of compounds from the specific etiologies, pathogeneses, and treatment methods.

Gao predicted that the main active ingredients in the antidepressant effect such as saikosaponin A, glycyrrhizin, ferulic acid, paeoniflorin, 6-gingerol, and atractylaractone I can play an antidepressant role by regulating Htr2a, Nmdar1, Pkc, CamkII, and Caspase-3 proteins in the glutaminergic synaptic pathway (Gao, 2021). Through TCSMP, PubChem, OMIM, and other databases, researchers found that paeoniflorin, quercetin, catechin, kaempferol, and other components in Xiaoyao Powder played an antidepressant role by acting on SRC, STAT3, JUN, MAPK3, and other targets (Jian et al., 2021). It has also been found that quercetin, kaempferol, and aloe emodin in Xiaoyao Powder can reduce the expression of inflammatory cytokines by acting on targets likes IL-4 and IL-6 to treat depression (Wei et al., 2021).

### Quality markers based on component measurability

Xu used UPLC-MS/MS to determine the content of paeoniflorin, ferulic acid, quercetin, isolicorice, atractylolactone III, saikosaponin A, saikosaponin C, and 14 other components in Xiaoyao Powder (Xu et al., 2018). Tian et al. (2015) used UPLC-DA to determine the contents of ligustilide, atractylenolide I, and atractylenolide II in Xiaoyao Powder.

**FIGURE 2:**
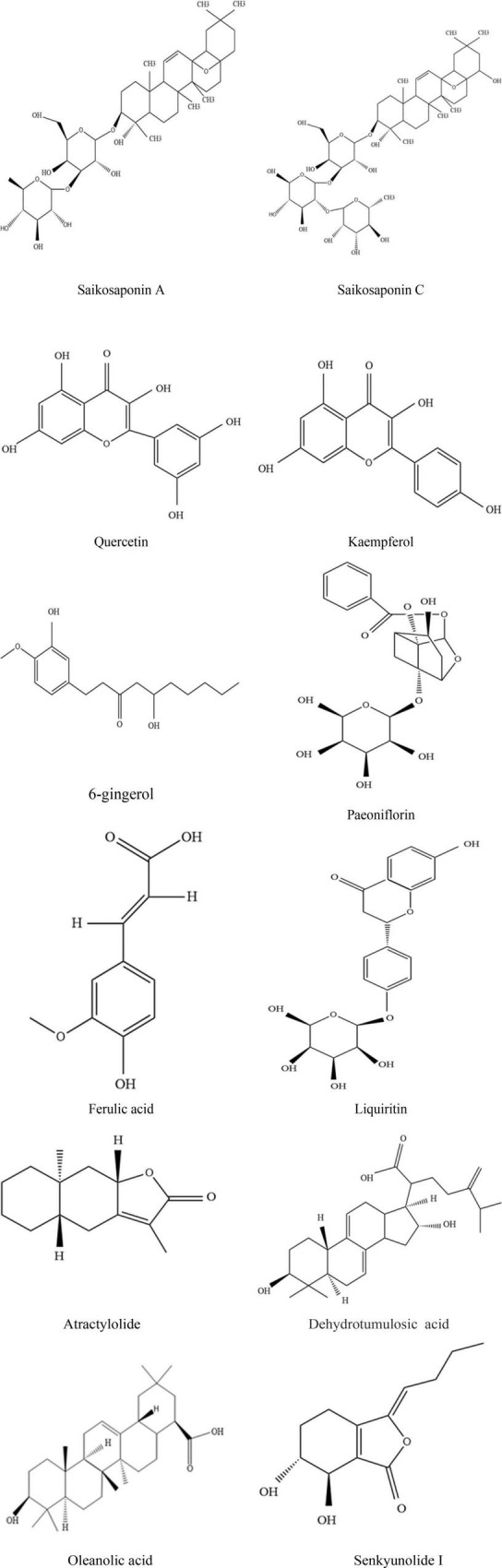
Molecular structure information of quality markers.

In summary, based on the “five principles” analysis of quality markers, it can be seen that saikosaponin A, saikosaponin C, quercetin, paeoniflorin, ferulic acid, glycyrrhizin, atractylolactone, dehydrolimoic acid, oleanolic acid, kaempferol, senkyunolide, and 6-gingerol are important quality markers in Xiaoyao Powder (Table 3 and Figure 2). They are highly specific, measurable and convenient for quality control, and can be used as quality markers for Xiaoyao Powder in the treatment of depression.

**TABLE 3:** Quality marker information.

Chemical compound	Molecular formula	CAS	Relative molecular mass	Source
Saikosaponin A	C_42_H_68_O_13_	20736-09-8	780.98	*Bupleurum Chinese* DC.
Saikosaponin C	C_48_H_78_O_17_	20736-08-7	927.12	*Bupleurum Chinese* DC.
Quercetin	C_15_H_10_O_7_	117-39-5	302.23	*Bupleurum Chinese* DC. and *Glycyrrhiza uralensis* Fisch.
Kaempferol	C_15_H_10_O_6_	520-18-3	286.24	*Bupleurum Chinese* DC.
Paeoniflorin	C_23_H_28_O_11_	23180-57-6	480.5	*Paeonia lactiflora* Pall.
Ferulic acid	C_10_H_10_O_4_	1135-24-6	194.18	*Angelica sinensis* (Oliv.) Diels.
Liquiritin	C_21_H_22_O_9_	551-15-5	418.4	*Glycyrrhiza uralensis* Fisch.
Atractylolide	C_15_H_18_O_2_	81130-16-7	230.32	*Atractylodes macrocephala* Koidz.
Dehydrotumulosic acid	C_15_H_10_O_7_	6754-16-1	484.71	*Poria cocos* (Schw.) Wolf.
Oleanolic acid	C_30_H_48_O_3_	508-02-1	456.7	*Poria cocos* (Schw.) Wolf.
6-gingerol	C_17_H_26_O_4_	23513-14-6	294.4	*Zingiber officinale* Rosc.
Senkyunolide I	C_12_H_16_O_4_	94596-28-8	224.253	*Angelica sinensis* (Oliv.) Diels.

## Summary

Depression is a common mental disorder caused by trauma and other factors, which is manifested by clinical manifestations such as low mood and loss of interest (Orsolini et al., 2019). At present, there are many drugs to treat depression, such as fluoxetine, propranolol, paroxetine, and other drugs. But the side effects are serious and many people do not have an effective solution for life and health. The classic famous prescription Xiaoyao Powder is the traditional prescription for treating depression. It can reduce side effects. Pharmacologically, It can adjust the HPA axis, regulate neuronal, and synaptic plasticity, regulate intestinal flora disorders, regulate neurotransmitters, receptors, and inflammation cytokines, and regulate DNA methylation. And twelve quality markers like Saikosaponin A, Quercetin, etc. are summarized. With the development of the times, the pharmacological mechanism has been deepening. More and more scientists have strengthened research on the microscopic molecular mechanism. There are also many clinical studies on the treatment of depression, especially the addition or subtraction of drugs based on the original prescription according to the different clinical manifestations of patients. However, there are few studies on the chemical composition of Xiaoyao Powder, and in particular, there is a lack of information on quality markers. It is hoped that scientists need to strengthen more detailed studies on the aspect in the future.

## Author contributions

JW: writing—original draft preparation. QW and CO: writing—review. GL and HY: writing—review and revise. All authors contributed to the present research and reviewed the entire manuscript, read, and agreed to the published version of the manuscript.

## Footnotes

1
https://tcmsp-e.com/
